# Author Correction: Extracting Intercellular Signaling Network of Cancer Tissues using Ligand-Receptor Expression Patterns from Whole-tumor and Single-cell Transcriptomes

**DOI:** 10.1038/s41598-018-36408-x

**Published:** 2018-12-12

**Authors:** Joseph X. Zhou, Roberto Taramelli, Edoardo Pedrini, Theo Knijnenburg, Sui Huang

**Affiliations:** 1 Institute for Systems Biology, Seattle, WA USA; 2 Department of Biotechnology and Molecular Science, University of Insubria, Varese, Italy

Correction to: *Scientific Reports* 10.1038/s41598-017-09307-w, published online 18 August 2017

This Article contains an error in the order of the Figures. Figures 5 and 6 were published as Figures 6 and 5 respectively. The correct Figures 5 and 6 appear below as Figures 1 and 2. The Figure legends are correct.

**Figure 1:**
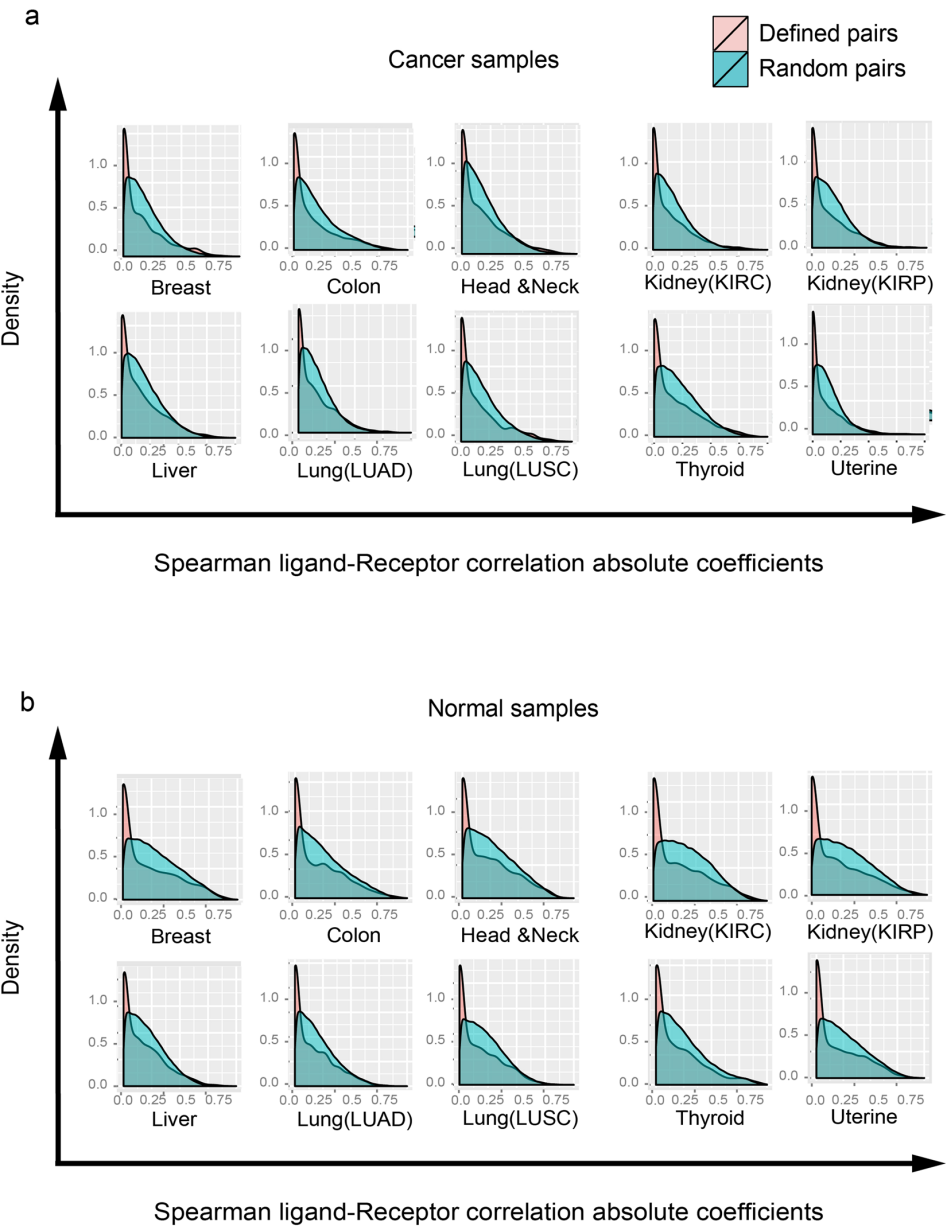
Spearman correlations in cancer and normal tissues are significantly different from randomly chosen pairs. (**a**) The Spearman correlation coefficients distribution (random (in red color) vs. defined (in blue) ligand-receptor gene pair) in cancer tissues. (**b**) The Spearman correlation coefficients distribution (random vs. defined ligand-receptor gene pair) in normal tissues.

**Figure 2:**
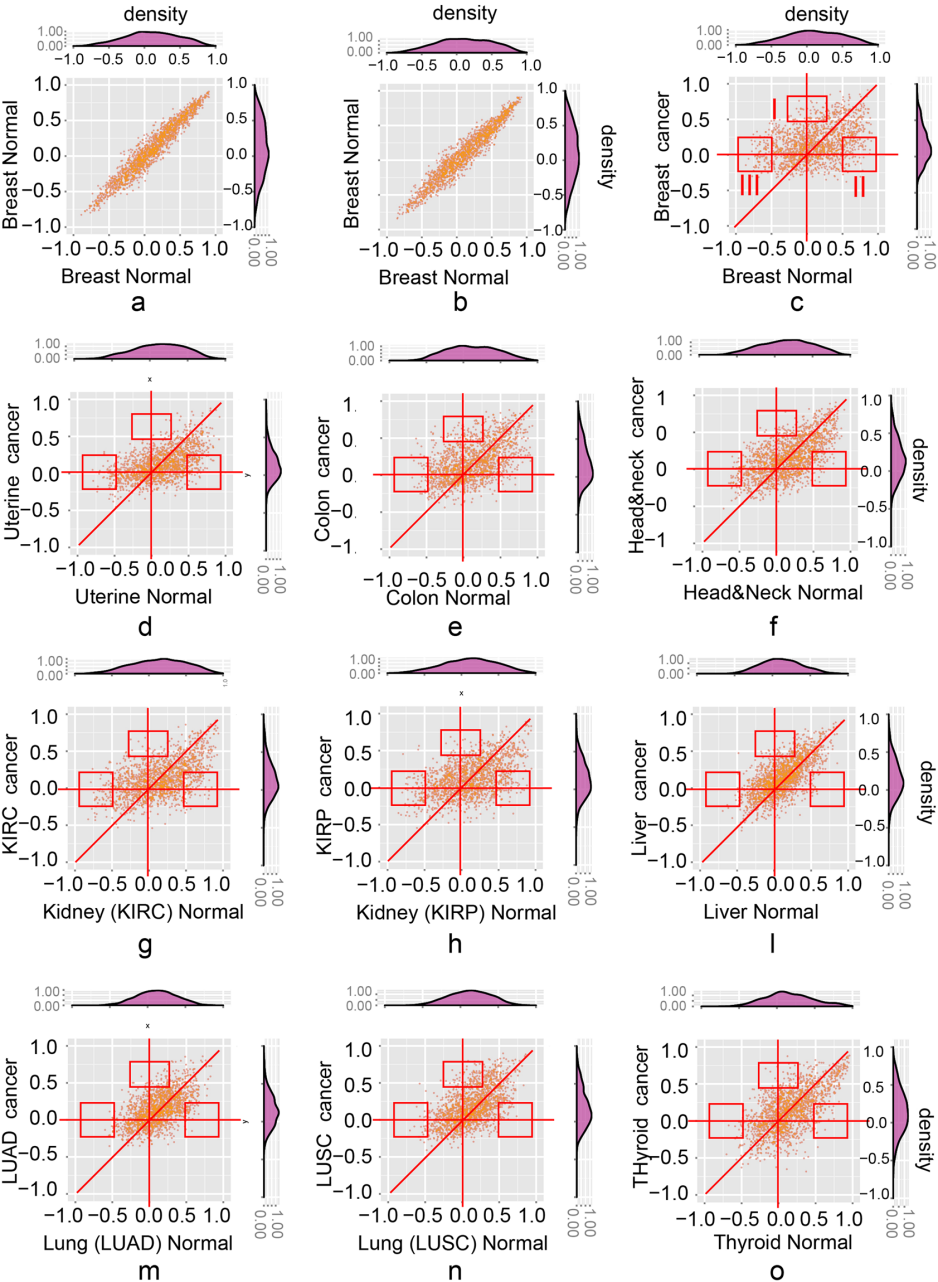
The scatter plots show the altered ligand-receptor correlations between normal and cancer tissues of 10 cancer types. (**a**,**b**) The background noise of correlations in the normal breast tissue. The scatter plots of 2,558 ligand-receptor pairs from 65 samples which are randomly chosen 1,000 times from breast tissue (performed twice); (**c**–**o**) The scatter plots of ligand-receptor correlations (normal vs. cancer) of ten cancer types. Three types of altered correlations: (i) area *I*: changing from uncorrelated (Spearman correlation coefficients between −0.25 and 0.25) to correlated (higher than 0.5); (ii) area *II*: changing from positively correlated (lower than −0.5) to uncorrelated (between −0.25 and 0.25); (iii) area *III*: changing from negatively correlated (higher than 0.5) to uncorrelated (between −0.25 and 0.25).

