# Supplementary material for: Extracting Intercellular Signaling Network of Cancer Tissues using Ligand-Receptor Expression Patterns from Whole-tumor and Single-cell Transcriptomes
Source: Sci Rep. 2017 Aug 18;7:8815. doi: 10.1038/s41598-017-09307-w (PMC5562796; doi:10.1038/s41598-017-09307-w)
Supplement: Supplementary file 1 — Supplemental material [file 41598_2017_9307_MOESM1_ESM.pdf]

Extracting Intercellular Signaling Network of Cancer Tissues using Ligand-Receptor Expression Patterns from Whole-tumor and Single-cell Transcriptomes

Joseph X. Zhou[1§], Roberto Taramelli[2§], Edoardo Pedrini[2], Theo Knijnenburg[1], Sui Huang[1*]

Affiliations:

[1] Institute for Systems Biology, Seattle WA, USA.

[2] Department of Biotechnology and Molecular Science, University of Insubria, Varese, Italy

[§] authors contributed equally as first author

*Correspondence to: sui.huang@systemsbiology.org

Supplementary

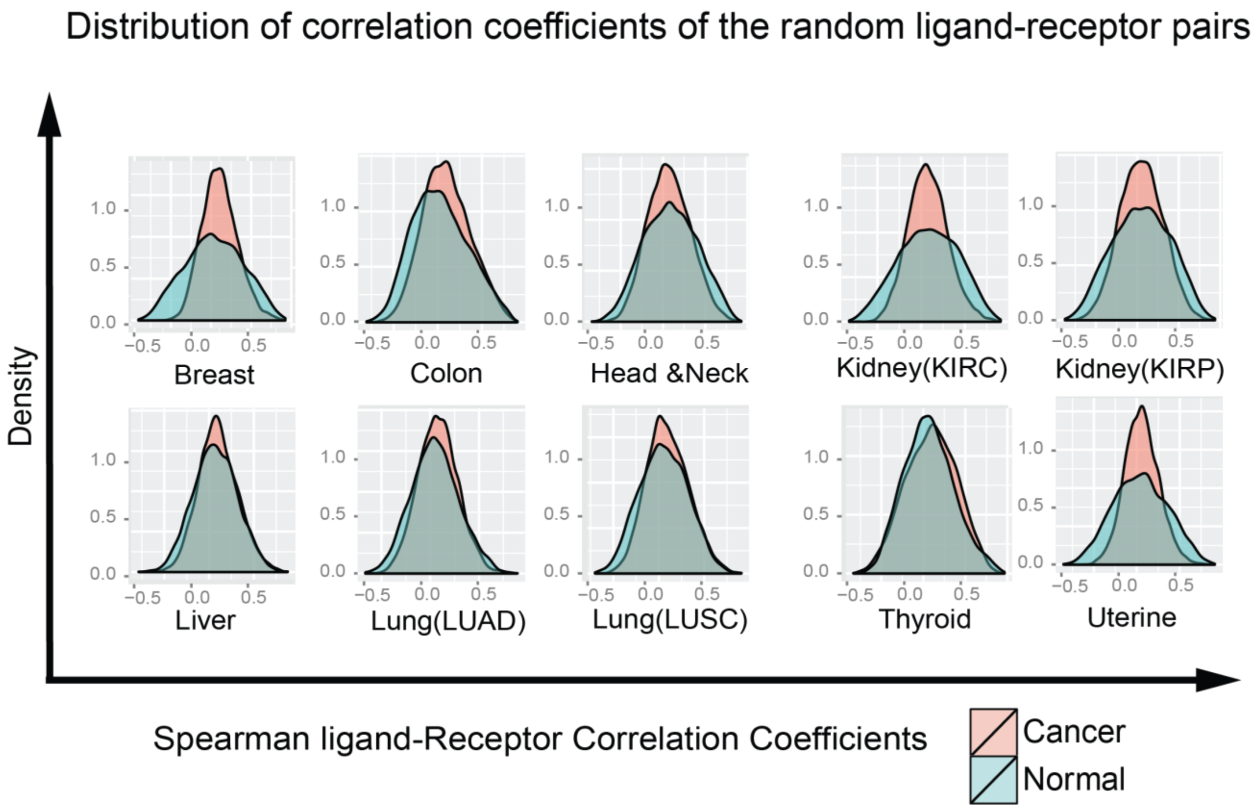

Figure S1. The distribution of correlation coefficients of the random ligand-receptor pairs for the samples of ten cancer types and their corresponding normal tissues. The random receptor/ ligand pairs were less correlated than the specific pairs at the population level for ten cancer types (see Fig. 4A).

No.	ligand	receptor	Shared cancer types number
1	COL11A1	ITGA2	5
2	COL7A1	ITGA2	5
3	MDK	GPC2	5
4	MMP1	ITGA2	5
5	COL1A1	ITGA11	4
6	COL1A1	ITGA2	4
7	CXCL5	CXCR2P1	4
8	FN1	PLAUR	4
9	JAG2	NOTCH3	4
10	MMP9	EPHB2	4
11	COL1A1	CD44	3
12	COL1A1	TMPRSS6	3
13	COL4A1	ITGA2	3
14	EFNA3	EPHA1	3
15	EFNA4	EPHA1	3

Table S1. The top 15 ligand-receptor pairs both are significantly up-regulated in caner tissues (LIMMA, two folds change, P-value=0.05)

No.	Ligand	Receptor	Ligand Change	Receptor Change	Shared cancer types number
1	CALM1	HMMR	0	1	9
2	SPP1	ITGAV	1	0	9
3	SPP1	ITGB1	1	0	9
4	TNFSF18	TNFRSF18	0	1	9
5	COL11A1	ITGB1	1	0	8
6	CTHRC1	FZD6	1	0	8
7	EFNA3	EPHA5	1	0	8
8	HSPA1A	GRIN2D	0	1	8
9	SPP1	ITGA5	1	0	8
10	ADAM12	ITGB1	1	0	7
11	ASIP	MC1R	0	1	7
12	COL11A1	DDR1	1	0	7
13	COL1A1	ITGAV	1	0	7
14	COL1A1	ITGB1	1	0	7
15	COL5A1	ITGB1	1	0	7

Table S2. The top 15 ligand-receptor pairs in which only one of them is significantly up-regulated in caner tissues (LIMMA, 1 means the gene has two folds up change with P-value =0.05, 0 means the gene unchanged)

No.	ligand	receptor	Shared cancer types
1	ANGPTL1	TEK	8
2	CCL21	CCBP2	7
3	ANGPT1	TEK	6
4	CCL14	CCBP2	6
5	L1CAM	CNTN1	6
6	NCAM1	GFRA1	6
7	ANGPT1	TIE1	5
8	APOD	LEPR	5
9	BST1	CAV1	5
10	CD34	SELP	5
11	CTGF	ERBB4	5
12	DCN	ERBB4	5
13	FGF7	FGFR2	5
14	L1CAM	FGFR2	5
15	LPL	GPIHBP1	5

Table S3. The top 15 ligand-receptor pairs both are significantly down-regulated in caner tissues (LIMMA, two folds change, P-value =0.05)

No.	Ligand	Receptor	Ligand Change	Receptor Change	Shared cancer types number
1	CLCF1	CNTFR	0	-1	9
2	CNTF	LIFR	0	-1	9
3	CORT	SSTR1	0	-1	9
4	CXCL12	CXCR4	-1	0	9
5	NPY	NPY1R	0	-1	9
6	RELN	ITGB1	-1	0	9
7	RSPO3	FZD8	-1	0	9
8	TGFB3	TGFBR3	0	-1	9
10	ANGPT4	TEK	0	-1	8
11	CCL21	CXCR3	-1	0	8
14	CXCL12	CXCR3	-1	0	8
16	GH1	GHR	0	-1	8
17	GH2	GHR	0	-1	8
18	HK1	TACR1	0	-1	8
20	IGF1	IGF1R	-1	0	8
21	LAMA2	ITGB1	-1	0	8
22	NCAM1	PTPRA	-1	0	8
23	PTN	CDH10	-1	0	8
24	UBA52	AGTR1	0	-1	8
27	CALM1	VIPR1	0	-1	7
28	CALM3	PDE1A	0	-1	7
29	CCL14	CCR1	-1	0	7
30	CCL14	CCR3	-1	0	7
31	CCL14	CCR5	-1	0	7
32	CCL25	CCBP2	0	-1	7
33	CCL27	CCBP2	0	-1	7
34	CCL3L3	CCBP2	0	-1	7
35	CCL7	CCBP2	0	-1	7
36	CNTF	IL6ST	0	-1	7

38	COL4A4	ITGAV	-1	0	7
39	COL4A4	ITGB1	-1	0	7
40	COL4A6	CD47	-1	0	7
41	COL4A6	ITGAV	-1	0	7
42	COL4A6	ITGB1	-1	0	7
45	CXCL12	CCR4	-1	0	7

Table S4. The top 45 ligand-receptor pairs in which only one of them is significantly down-regulated in caner tissues (LIMMA, -1 means the gene has two folds down change with P-value =0.05, 0 means the gene unchanged)

No.	Code	Full Name	Distance	P-value
1	*BRCA*	*Breast Carcinoma*	*0.18*	*0*
2	*COAD*	*Colon adenocarcinoma*	*0.17*	*0*
3	*HNSC*	*Head & Neck squamous cell carcinoma*	*0.10*	*1.24E-08*
4	*KIRC*	*Kidney renal clear cell carcinoma*	*0.14*	*3.77E-15*
5	*KIRP*	*Kidney renal papillary cell carcinoma*	*0.14*	*1.72E-14*
6	*LIHC*	*Liver hepatocellular carcinoma*	*0.08*	*2.65E-05*
7	*LUAD*	*Lung adenocarcinoma*	*0.09*	*1.94E-06*
8	*LUSC*	*Lung squamous cell carcinoma*	*0.11*	*2.11E-09*
9	*THCA*	*Thyroid carcinoma*	*0.03*	*0.24*
10	*UCEC*	*Uterine Corpus Endometrial Carcinoma*	*0.16*	*0*

Table S5. *Kolmogrov-Sminov* test of *Spearman* correlation distribution between normal and caner tissues. The distances and P-values of *Spearman* correlation coefficients distribution between 10 normal and cancer tissues from TCGA were calculated from *Kolmogrov-Sminov* tests. The P-value of 0.01 is set as the cut-off for the significant change of two distributions.

No.	ligand	receptor	Shared cancer types
1	*CCL2*	*CCR5*	5
2	*C1QA*	*CR1*	4
3	*CCL3*	*CCR5*	4
4	*ICAM1*	*ITGAM*	4
5	*IL15*	*IL2RB*	4
6	*PLAU*	*ITGA5*	4
7	*VCAM1*	*ITGB7*	4
8	*B2M*	*LILRB1*	3
9	*CCL2*	*CCR2*	3
10	*CCL4*	*CCR5*	3
11	*CCL8*	*CCR5*	3
12	*CSF1*	*CSF1R*	3
13	*CXCL12*	*CD4*	3
14	*FN1*	*ITGAV*	3
15	*GNAI2*	*FPR1*	3

Table S6. The top 15 ligand-receptor pairs change most from uncorrelated in normal (the Spearman correlations are between -0.25 and 0.25) to correlated (*Spearman* correlations are higher than 0.5) in caner tissues

No.	ligand	receptor	Shared cancer types
1	*CGN*	*TGFBR2*	5
2	*CHAD*	*ITGB1*	5
3	*SEMA6D*	*TYROBP*	5
4	*IHH*	*BOC*	4
5	*VEGFA*	*EPHB2*	4
6	*COL4A1*	*CD47*	3
7	*FGF2*	*FGFR4*	3
8	*FGF2*	*SDC1*	3
9	*GNAI2*	*IGF1R*	3
10	*GNAS*	*ADCY7*	3
11	*INHA*	*ACVR1*	3
12	*LAMA1*	*NT5E*	3
13	*PIGF*	*FLT1*	3
14	*PSAP*	*CELSR1*	3
15	*RTN4*	*TNFRSF19*	3

Table S7. The ligand-receptor pairs change most from negatively correlated in normal (Spearman correlations are lower than -0.5) to uncorrelated (Spearman correlations are between -0.25 and 0.25) in caner tissues

No.	ligand	receptor	Shared cancer types
1	*SEMA4G*	*PLXNB2*	5
2	*EFNB3*	*RHBDL2*	4
3	*FGF2*	*NRP1*	4
4	*LIPH*	*LPAR2*	4
5	*TGFB3*	*TGFBR2*	4
6	*ANGPT1*	*ITGB1*	3
7	*APOE*	*CHRNA4*	3
8	*BMP6*	*BMPR2*	3
9	*CDH1*	*ERBB3*	3
10	*CDH1*	*PTPRF*	3
11	*COL5A3*	*SDC3*	3
12	*EFNA3*	*EPHA1*	3
13	*EFNA3*	*EPHB6*	3
14	*F8*	*LRP1*	3
15	*FGF7*	*NRP1*	3

Table S8. The ligand-receptor pairs change most from positively correlated in normal (Spearman correlations are higher than 0.5) to uncorrelated (Spearman correlations are between -0.25 and 0.25) in caner tissues
